# Postoperative proximal junctional kyphosis correlated with thoracic inlet angle in Lenke 5c adolescent idiopathic scoliosis patients following posterior surgery

**DOI:** 10.1186/s12891-022-05868-8

**Published:** 2022-10-17

**Authors:** Bowen Hu, Linnan Wang, Yueming Song, Xi Yang, Limin Liu, Chunguang Zhou

**Affiliations:** Department of Orthopedic Surgery and Orthopedic Research Institute, West China Hospital, Sichuan University, No. 37 GuoXue Road, Chengdu, Sichuan China

**Keywords:** Proximal junctional kyphosis, Thoracic inlet angle, Adolescent idiopathic scoliosis, Sagittal alignment, Thoracolumbar/lumbar curve

## Abstract

**Background:**

Proximal junctional kyphosis is a common complication after posterior fusion in patients with adolescent idiopathic scoliosis and is correlated with postoperative changes of thoracic kyphosis. In lenke 5c patients, higher postoperative LL and spontaneous change of TK may produce an effect on final PJK. However, no studies has been performed to evaluate the correlation of PJK with thoracocervical parameters in patients with AIS.

**Methods:**

Data from 98 patients who underwent posterior fusion for Lenke 5C AIS with 2 years of follow-up were retrospectively reviewed. Patients in the extended fusion group underwent fusion at levels higher than upper-end vertebra + 2 (*n* = 38), and those in the thoracolumbar/lumbar (TL/L) fusion group underwent fusion at UEV + 2 or lower (*n* = 60).

**Results:**

During an average follow-up of 38.1 months, 23 of 98 patients developed PJK. The extended fusion group had a higher incidence of PJK than the TL/L fusion group (14/38 vs. 9/60, respectively; *P* = 0.01) and a significantly greater decrease in thoracic kyphosis than the TL/L group (*P* < 0.01). Patients with PJK had a significantly larger preoperative thoracic inlet angle (TIA) than those without PJK (*P* < 0.01). Multivariate analysis showed that a greater preoperative TIA and extended fusion were associated with PJK. The Scoliosis Research Society 22-item questionnaire score did not significantly differ between the PJK and non-PJK groups.

**Conclusions:**

The preoperative TIA could be a predictor of PJK. Among patients with Lenke 5C AIS, those with a TIA of > 71° are more likely to develop PJK. Additionally, extended fusion in patients with Lenke 5C may increase the risk of PJK.

## Introduction

Proximal junctional kyphosis (PJK) is a common complication after posterior fusion in patients with Lenke 5C adolescent idiopathic scoliosis (AIS) [1–4]. Multiple studies have shown that the risk factors for PJK are a large lumbar lordosis (LL) angle, large sagittal vertical axis (SVA), and low pelvic incidence (PI) [1–4]. Other factors strongly associated with PJK in patients with AIS are large preoperative thoracic kyphosis (TK) and large immediately postoperative TK [2, 4, 5]. Previous studies have indicated that the thoracic inlet angle (TIA) is strongly associated with TK and global TK (GTK) in patients with AIS [6–8] and that the TIA is a constant morphological parameter that is not changed by the patient’s position or any other conditions. Additionally, these correlations are maintained at 2 years after corrective surgery in patients with Lenke 1 AIS [8]. Hence, the TIA is an ideal parameter that can be used to evaluate the sagittal balance of the proximal thoracic area and may be a predictor of PJK.

The choice of the uppermost instrumented vertebra (UIV) may also be associated with the incidence of PJK in patients with Lenke 5C AIS [4]. Anterior selective thoracolumbar correction and fusion were widely used in the Lenke 5c patients, which might hardly correct the unfused thoracic curve [9]. Meanwhile, posterior thoracolumbar/lumbar (TL/L) fusion is commonly performed to treat Lenke 5C AIS nowadays [10, 11]. To avoid progression of the unfused thoracic curve after TL/L fusion, experienced surgeons may choose to perform extended fusion in patients with Lenke 5C AIS with a bending thoracic Cobb angle of > 20° [12–15]. Furthermore, Kwan et al. [16] suggested the performance of extended fusion in patients with a bending thoracic curve of > 15°. However, extended fusion may strongly reduce the TK in the sagittal profile [15, 16]; reduction of TK is a proven risk factor for PJK and may also result in great changes in thoracocervical parameters in patients with AIS [2, 4, 5, 17–21].

The present study focused on analysis of the radiological features of patients with Lenke 5C AIS to identify the correlations among the TIA, PJK, and other sagittal parameters. We also evaluated the relationship between the choice of the UIV and PJK in patients with Lenke 5C AIS.

## Materials and methods

The present retrospective study included 98 patients with Lenke 5 AIS from January 2010 to January 2019 who underwent posterior-only surgery in the department of Orthopedic Surgery, West China hospital, Sichuan university. All included patients underwent follow-up for more than 2 years with complete radiographic data. The exclusion criteria were incomplete follow-up data; poor radiographic images; age > 20 years; follow-up period < 2 years; and a history of spine surgery. The present study was approved by the local ethics committee. Patients in the extended fusion group underwent fusion higher than two levels above the upper-end vertebra, whereas patients in the TL/L fusion group underwent fusion below or at the upper-end vertebra + 2. Patients with a Cobb angle of the thoracic curve of > 40° or a bending thoracic curve of > 20° generally underwent extended fusion; however, a few patients with a rigid thoracic curve chose to undergo TL/L fusion to preserve the flexibility of the thoracic spine after acknowledging complete understanding of the benefits and risks of these two procedures. The bending thoracic curve of > 20° and rib hump were prognostic factors of higher final thoracic curves angle [22]. However, no ribs were resected because of the mild rib hump in the lenke 5c patients.

All surgeries were performed by the same surgical team with two senior surgeons. Supraspinous and interspinous ligaments were removed. Smith-Petersen osteotomy was performed before fusion. The Legacy or CD Horizon M8 screw-rod system (Medtronic Sofamor Danek, Inc., Memphis, TN, USA) was used for fixation (5.5 mm titanium rod). Polyaxial screws were inserted in the UIV and LIV. The monoaxial screws were mostly used in the other vertebrae. Rod rotation, apical vertebral derotation (by vertebral column manipulation or direct vertebral rotation), convex compression, and concave distraction were used intraoperatively to correct scoliosis and rotation of the lumbar spine.

Standing full-length posteroanterior and lateral radiographs were routinely taken using a multipurpose digital radiographic fluoroscopy system (SONIALVISION safire 17; Shimadzu Corporation, Kyoto, Japan) preoperatively, at 3 months postoperatively, and at the final follow-up. Radiologic parameters were independently measured and documented on an electronic system by two surgeons who were not involved in the surgeries, and the average values were calculated to increase the accuracy of the measurement of the upper thoracic region [20].

**Fig. 1:**
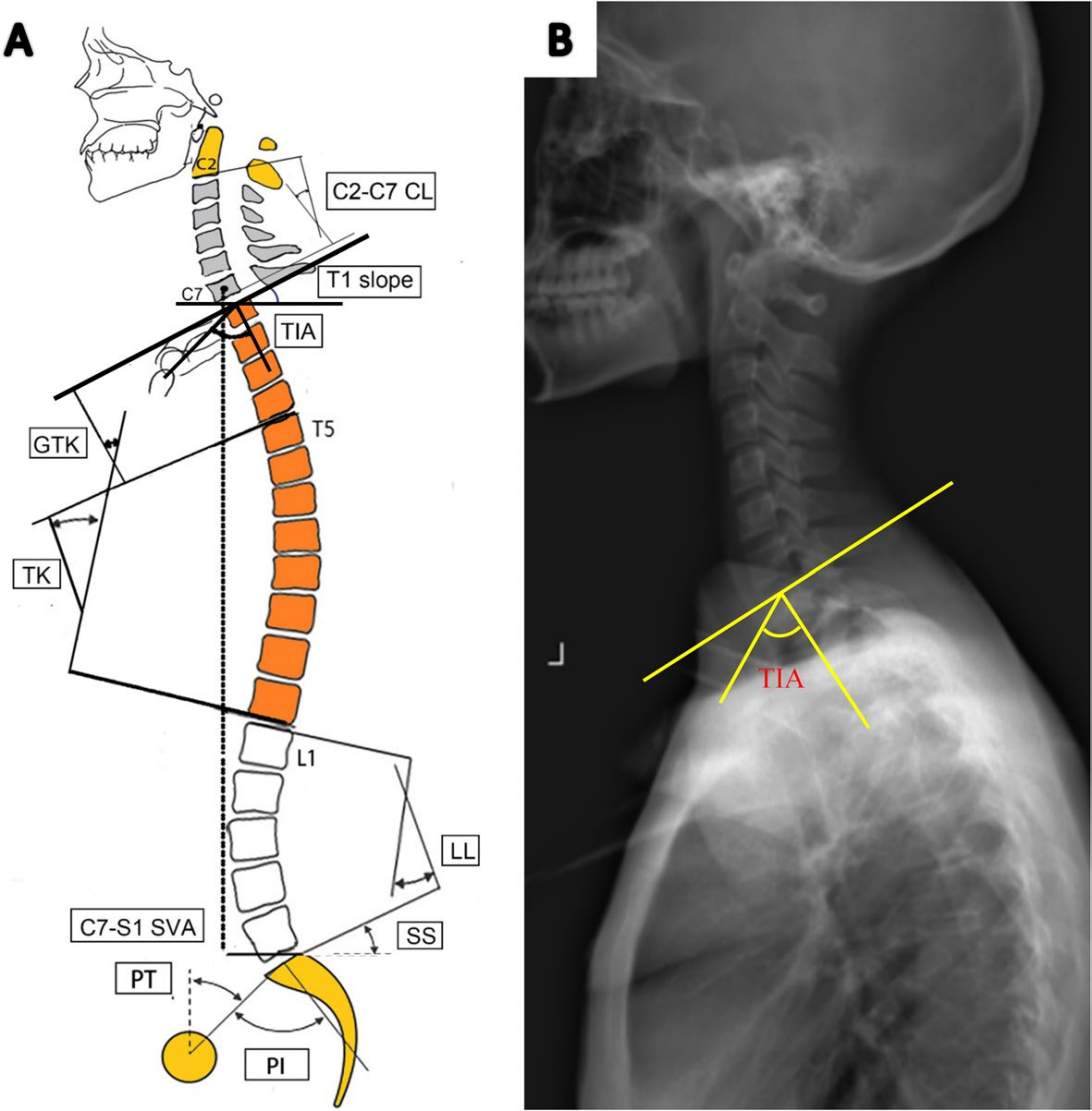
**A** Spinal parameters in standing lateral radiographs. T means Thoracic vertebra. TIA means Thoracic inlet angle. LL means Lumbar lordosis. TK means thoracic kyphosis. GTK means global thoracic kyphosis. PI means Pelvic incidence. PT means Pelvic tilt. SS means Sacral slope. SVA means Sagittal vertical axis. **B** Figure about measurement of TIA

The coronal parameters measured were the main TL/L Cobb angle, thoracic Cobb angle, reduced bending angle, and correction rate of the two curves. The sagittal parameters measured were the PI, sacral slope (SS), pelvic tilt (PT), LL, PI-LL, TK, GTK, T1 tilt, TIA, proximal junctional angle (PJA), and SVA. TK, GTK, TK, LL, PI, SS, and PT were measured using previously described standard methods (Fig. 1).

The TIA was defined as the angle between the vertical line of the T1 superior endplate and the line passing through the midpoint of T1. The PJA was defined as the Cobb angle between the lower endplates of the UIV and the upper endplates of the two supra-adjacent vertebrae. PJK was defined by the following two criteria with a ≥ 2-year follow-up time: (1) final PJA of ≥ 10° and (2) final PJA at least 10° greater than the preoperative measurement [1, 2, 4, 5]. The presence of both criteria was necessary for considering PJK. An increase in the PJK angle was defined as an increase from the preoperative time to the last follow-up time. Clinical assessments were made using the Scoliosis Research Society 22-item questionnaire (SRS-22).

### Statistical analysis

Student’s *t*-test was performed to analyze the postoperative changes in the Cobb angle of the thoracic and TL/L curves, PI, PT, SS, SVA, TK, GTK, PI-LL, TIA, and T1 tilt. Bivariate correlation tests, Student’s *t*-test, the chi-squared test, and receiver operating characteristic (ROC) curve estimations were performed to determine the changes in sagittal balance from the preoperative period to the final follow-up. Data were analyzed using SPSS 21.0 statistical software (IBM Corp., Armonk, NY, USA). To investigate the influence of preoperative measurements on postoperative outcomes, certain preoperative variables (PI, PT, SS, SVA, PJA, PI-LL, GTK, TIA, and T1 tilt) and type of fusion (selective or extended) were selected for inclusion in the multivariate analysis based on the results of the correlation analysis and clinical knowledge. Statistical significance was defined as *p* < 0.05.

## Results

The mean age of the 98 patients with Lenke 5 AIS in this study was 15.6 ± 2.6 years. The patients comprised 77 females and 21 males, and the mean Risser sign was 3.4 ± 1.5. The mean follow-up duration was 38.1 months (range, 24–72 months). The mean Cobb angle of the main TL/L curve was 53.2° ± 9.1° before surgery and 9.4° ± 7.5° at the final follow-up.

Sixty patients underwent selective TL/L fusion, and 38 patients underwent extended fusion. Both groups had an excellent correction rate for the main TL/L curve at the final follow-up (82.6% in the extended fusion group and 82.2% in the TL/L fusion group). The correction rate of the thoracic curve in the extended fusion group (71.9%) was greater than that in the TL/L fusion group (52.6%).

The PI and TIA remained stable after surgery and throughout follow-up in both the extended fusion and TL/L fusion groups. No significant difference in the PI, SS, PT, LL, or SVA were found between the TL/L fusion and extended fusion group preoperatively or at the final follow-up(Table 1). A significant difference was found in the immediate change of TK (− 1.4 ± 11.1 vs. 2.9 ± 6.0, *p* = 0.01) and in the PJK angle (8.2° ± 7.7° vs. 4.4° ± 4.9°, *p* = 0.01) between the extended fusion and TL/L fusion groups.

**Table 1:** The comparison between extended fusion and TL/L fusion group

Variables	Extended fusion group (*n* = 38)	TL/L fusion group (*n* = 60)	*P* value
Age (years)	15.4 ± 2.5	15.7 ± 2.7	0.66
Risser sign	3.2 ± 1.4	3.4 ± 1.6	0.54
Pre TK form (T5-T12)			
Hyperkyphosis	4	2	
Normal	30	50	
Hypokyphosis	4	8	
Mean fusion level	11.2 ± 1.2	7.1 ± 1.1	< 0.01*
Cobb angle of main TL/L curve(°)			
Pre-OP	54.9 ± 8.9	52.1 ± 9.1	0.14
Bending	26.3 ± 10.2	25.3 ± 10.2	0.65
Post-OP	8.5 ± 7.1	8.0 ± 7.0	0.72
Follow-Up	9.6 ± 7.7	9.3 ± 7.5	0.85
Cobb angle of T curve(°)			
Pre-OP	36.4 ± 7.3	26.5 ± 6.8	< 0.01*
Bending	21.8 ± 3.3	13.8 ± 6.6	< 0.01*
Post-OP	9.6 ± 6.4	11.0 ± 7.4	0.35
Follow-Up	10.2 ± 6.3	12.6 ± 8.1	0.13
TK(°)			
Pre-OP	22.6 ± 12.5	19.6 ± 8.8	0.16
Post-OP	21.2 ± 8.3	22.5 ± 7.6	0.40
Follow-Up	22.3 ± 11.3	24.9 ± 8.5	0.21
Immediate Post-OP change	-1.4 ± 11.1	2.9 ± 6.0	0.01*
GTK (°)			
Pre-OP	29.1 ± 13.6	28.3 ± 11.8	0.78
Post-OP	30.0 ± 9.2	31.8 ± 11.0	0.40
Follow-Up	31.8 ± 12.1	33.9 ± 10.8	0.36
LL (°)			
Pre-OP	52.8 ± 10.7	51.0 ± 11.3	0.42
Post-OP	52.1 ± 8.3	50.3 ± 9.4	0.33
Follow-Up	52.7 ± 9.8	52.1 ± 9.0	0.78
TIA(°)			
Pre-OP	67.2 ± 7.5	66.7 ± 7.2	0.77
Post-OP	67.3 ± 7.2	67.4 ± 7.0	0.94
Follow-Up	67.7 ± 7.5	66.7 ± 7.1	0.54
PJA(°)			
Pre-OP	8.9 ± 4.0	8.9 ± 4.4	0.97
Post-OP	12.8 ± 5.9	10.1 ± 5.2	0.02*
Follow-Up	17.1 ± 8.5	13.3 ± 6.5	0.01*
Increasing PJK	8.2 ± 7.7	4.4 ± 4.9	0.01*

Hyperkyphosis:TK > 40° Hypokyphosis:TK < 10° Normal: 10° ≤ TK ≤ 40°

*TL/L* means Thoracolumbar/lumbar curve, *T* means Thoracic, *TK* means Thoracic kyphosis. *GTK* means Global thoraicic kyphosis. *LL* means Lumbar lordosis, *TIA* means Thoracic inlet angle, *PJA* Proximal junctional angle, *PJK* means Proximal junctional kyphosis

^*^means *P* < 0.05

According to our PJK criteria, 23 (23.5%) of 98 patients were considered to have PJK at the final follow-up. We found no significant difference in the PI, PT, SS, LL, PI-LL, or even SVA preoperatively, at 3 months, and at the final follow-up. In addition, the PJA did not differ between the two groups preoperatively. However, the mean preoperative and final TIA,T1 tilt, TK and GTK values in patients with PJK were significantly larger than those in patients without PJK (Table 2). At the final follow-up, a greater proportion of patients in the extended fusion group had PJK than in the TL/L fusion group (14/38 vs. 9/60, *p* < 0.01). The preoperative TIA of patients with PJK at the last follow-up was larger than that of patients without PJK (73.8° ± 5.0° vs. 64.8° ± 6.8°, *p* < 0.01), and the ROC curve showed that the cutoff value was 71°.

**Table 2:** The comparison between PJK and Non-PJK patients

Variables	PJK (*n* = 23)	Non-PJK (*n* = 75)	*P* value
Age (years)	15.3 ± 2.6	15.7 ± 2.6	0.49
Risser sign	2.9 ± 1.7	3.5 ± 1.5	0.08
Extended fusion	14/23	24/75	0.01
TK(°)			
Pre	27.4 ± 13.7	18.7 ± 8.3	< 0.01*
Post	26.6 ± 7.4	20.6 ± 7.5	< 0.01*
Follow	31.8 ± 10.2	21.5 ± 8.1	< 0.01*
GTK (°)			
Pre	35.8 ± 12.2	26.4 ± 11.8	< 0.01*
Post	37.7 ± 8.2	29.1 ± 10.1	< 0.01*
Follow	42.2 ± 9.2	30.3 ± 10.4	< 0.01*
TIA (°)			
Pre	74.6 ± 4.8	64.5 ± 6.2	< 0.01*
Post	73.5 ± 5.1	65.5 ± 6.5	< 0.01*
Follow	74.3 ± 5.0	64.9 ± 6.3	< 0.01*
T1 tilt(°)			
Pre	19.0 ± 8.3	14.0 ± 9.2	0.02*
Post	22.1 ± 7.5	15.0 ± 9.4	< 0.01*
Follow	24.2 ± 7.2	16.0 ± 8.5	< 0.01*
PJA (°)			
Pre	9.4 ± 4.1	8.7 ± 4.3	0.50
Post	16.7 ± 4.5	9.5 ± 4.7	< 0.01*
Follow	24.5 ± 6.6	11.8 ± 4.9	< 0.01*
Increasing PJK	15.1 ± 4.9	3.0 ± 3.4	< 0.01*
LL(°)			
Pre	55.2 ± 11.8	50.6 ± 10.7	0.09
Post	54.4 ± 9.1	50.0 ± 8.8	0.04*
Follow	57.1 ± 8.6	50.9 ± 9.0	0.01*
PI(°)			
Pre	46.1 ± 11.0	45.6 ± 9.7	0.84
Post	45.9 ± 10.9	45.8 ± 10.0	0.96
Follow	45.6 ± 10.7	45.6 ± 10.0	0.99
PT(°)			
Pre	6.5 ± 7.8	6.9 ± 7.4	0.80
Post	8.1 ± 8.6	9.2 ± 6.4	0.50
Follow	7.9 ± 7.8	8.8 ± 7.4	0.62
SS(°)			
Pre	39.6 ± 8.3	38.6 ± 7.7	0.61
Post	37.7 ± 7.4	36.6 ± 7.5	0.52
Follow	37.7 ± 7.7	36.9 ± 8.0	0.65
PI-LL(°)			
Pre	-9.1 ± 12.1	-5.1 ± 11.2	0.14
Post	-8.5 ± 11.0	-4.2 ± 9.0	0.06
Follow	-11.5 ± 9.8	-5.2 ± 9.5	0.01*
SVA (mm)			
Pre	-10.4 ± 36.0	-7.7 ± 25.3	0.69
Post	-4.2 ± 22.7	-8.3 ± 25.4	0.50
Follow	-9.3 ± 19.5	-8.2 ± 25.3	0.85

*TL/L* means Thoracolumbar/lumbar curve, *T* means Thoracic, *TK* means Thoracic kyphosis, *GTK* means Global thoraicic kyphosis, *TIA* means Thoracic inlet angle, *PJA* Proximal junctional angle, *PJK* means Proximal junctional kyphosis, *CL* means Cervical lordosis, *LL* means Lumbar lordosis, *PI* means Pelvic incidence, *PT* means Pelvic tilt, *SS* means Sacral slope, *SVA* means Sagittal vertical axis

^*^means *P* < 0.05

**Fig. 2:**
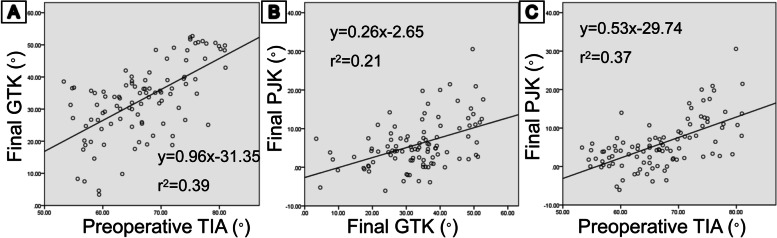
**a** Correlation of the preoperative TIA and the final GTK (*p* < 0.01). **b**, **c** Increasing PJK angle is highly correlated with the final GTK (*p* < 0.01) and preoperative TIA (*p* < 0.01)

A correlation analysis between each sagittal parameter and an increasing PJK angle was performed (Fig. 2). Bivariate correlation tests of several radiographic parameters showed that the preoperative TIA (*r* = 0.61, *p* < 0.001), preoperative TK (*r* = 0.44, *p* < 0.001), final TK (*r* = 0.52, *p* < 0.001), final T1 tilt (*r* = 0.43, *p* = 0.001), and final GTK (*r* = 0.46, *p* < 0.001) had a strong correlation with an increasing PJK angle. Preoperative GTK (*r* = 0.28, *p* = 0.006), preoperative T1 tilt (*r* = 0.27, *p* = 0.006), final LL (*r* = 0.30, *p* = 0.002), and final PI-LL (*r* = -0.25, *p* = 0.013) showed a weak correlation with an increasing PJK angle. No marked correlation was found in PI, PT, SS, SVA, or preoperative PI-LL.

The data suggested that the preoperative TIA could be a predictive parameter for the final sagittal balance. We also found that it had a correlation with the final T1 tilt (*r* = 0.59, *p* < 0.001), final TK (*r* = 0.45, *p* < 0.001), final GTK (*r* = 0.62, *p* < 0.001), increasing PJK angle (*r* = 0.61, *p* < 0.001), final PI-LL (*r* = -0.23, *p* = 0.02), and final LL (*r* = 0.32, *p* = 0.002). The final PI, PT, and SS had no significant correlation with the preoperative TIA.

The results of multivariate analysis for preoperative predictors of PJK are presented in Table 3. A greater preoperative TIA (*p* < 0.001) and extended fusion (*p* = 0.011) were found to be positive predictors of PJK.

**Table 3:** Multivariate analysis for preoperative factors associated with PJK In Lenke5c patients

	β	Standard Error	*P*	OR	95% CI for OR	
					Lower	Upper
Pre TIA	0.331	0.091	< 0.001*	1.393	1.165	1.664
Pre T1Stilt	-0.051	0.080	0.414	0.950	0.813	1.110
Pre GTK	0.116	0.086	0.174	1.123	0.950	1.328
Pre TK	0.077	0.090	0.395	1.080	0.905	1.288
Pre PJA	-0.174	0.115	0.131	0.841	0.671	1.053
Pre PI	-0.044	0.062	0.482	0.957	0.847	1.081
Pre SS	0.204	0.144	0.158	1.226	0.924	1.626
Pre LL	-0.169	0.126	0.179	0.845	0.660	1.081
Pre SVA	-0.271	0.246	0.271	0.763	0.471	1.235
Extended fusion	2.642	1.036	0.011*	14.037	1.843	106.938

*PJK* means Proximal junctional kyphosis, *TIA* means Thoracic inlet angle, *GTK* means Global thoraicic kyphosis, *TK* means Thoraicic kyphosis, *PJA* Proximal junctional angle, *LL* means Lumbar lordosis, *PI* means Pelvic incidence, *SS* means Sacral slope, *SVA* means Sagittal vertical axis

^*^means *P* < 0.05

Additionally, patients with PJK showed significantly greater LL and smaller PI-LL at the final follow-up time. No significant difference was found in the preoperative or final SRS-22 scores between postoperative patients with and without PJK (Table 4).

**Table 4:** Comparison of Scoliosis Research Society 22 Questionnaire between PJK and Non-PJK patients

	Pre-op		*P* value	follow-up		*P* value
	PJK	Non-PJK		PJK	Non-PJK	
Function/activity	4.1 ± 0.7	4.1 ± 0.8	0.84	4.0 ± 0.8	4.2 ± 0.6	0.15
Pain	4.1 ± 0.6	4.0 ± 0.4	0.38	4.4 ± 0.5	4.6 ± 0.4	0.12
Self-image	3.3 ± 0.7	3.5 ± 0.5	0.13	4.1 ± 0.6	4.1 ± 0.5	0.58
Mental health	3.6 ± 0.7	3.7 ± 0.6	0.47	4.4 ± 0.6	4.3 ± 0.6	0.36
Satisfication	3.8 ± 0.5	4.0 ± 0.6	0.10	4.2 ± 0.7	4.2 ± 0.6	0.70
Total score (w/o satisfaction questions)	3.8 ± 0.5	3.8 ± 0.4	0.52	4.2 ± 0.5	4.3 ± 0.4	0.39

No significant difference was found

## Discussion

Kim [1] and Wang [2] defined PJK in patients with AIS as both a > 10° increase in kyphosis between the UIV and the UIV + 2 and a final PJA of ≥ 10°. These criteria are widely used in patients with AIS [1, 2, 4, 5] and were chosen in our study. Sun et al. [3] reported that a > 10° increase in PJK occurred after selective fusion in 17.1% of patients with Lenke 5C AIS at 2 years postoperatively, which is comparable with the incidence of PJK found in the present study (23.5%).

Preoperative GTK and TK are alternate parameters that could potentially be used to predict the postoperative change in the sagittal balance of the thoracic spine. However, these parameters are easily influenced by posture because it is difficult to ensure that every patient has a horizontal vision line and is standing perfectly straight during radiography. Therefore, they are not ideal predictors of an increasing PJK angle. In contrast, the TIA is a constant morphological parameter that is not influenced by posture under any conditions, similar to the PI of the spinopelvic unit [6–8]. Recent studies have shown significant correlations between the preoperative TIA and GTK angle [6–8]. In our study, the TIA could be used to predict the GTK at the final follow-up, which was also strongly correlated with the final increasing PJK angle. Meanwhile, the occurrence of PJK is mainly determined by the variation in the TIA. This may explain the role of the TIA in predicting PJK.

**Fig. 3:**
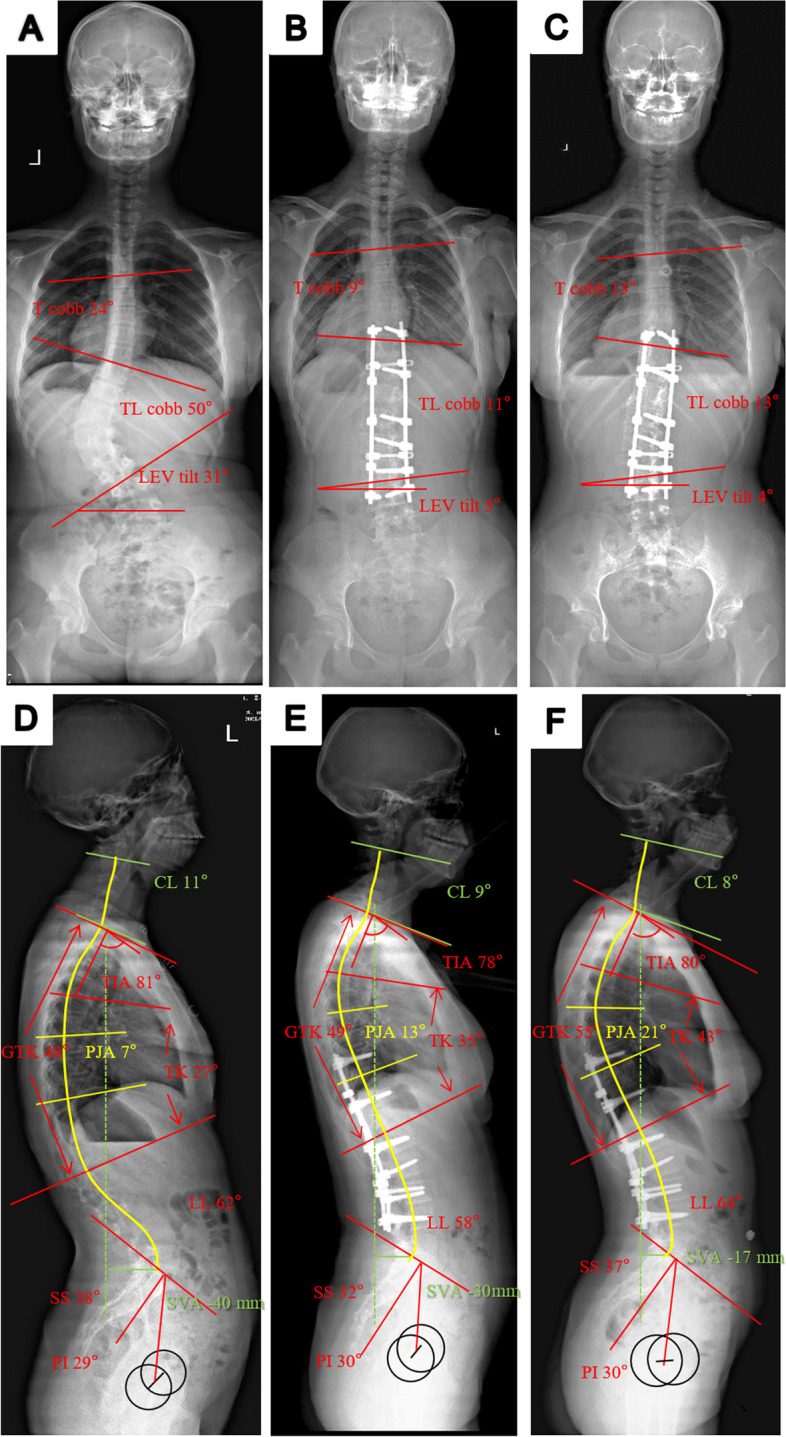
Standing PA and lateral radiographs of a 13-year-old female Lenke 5C patient with a structural thoracolumbar curve of 50° in TL/L fusion group. **a** Preoperative Cobb angle of thoracic curve was 24°, reduced to 13° at bending film. Preoperative LEV tilt was 31°. **b** The postoperative thoracic curve was corrected to 9° and thoracolumbar curve was corrected to 11° at first follow-up. **c** At 4-year follow-up, the main thoracolumbar curve was 13° and the thoracic curve was 13°. **d** The preoperative radiograph showed large TIA (81°) and GTK (48°). The PJA was 7° and TK was 27°. **e** At first follow-up: lateral radiograph illustrating stable TIA(78°) and GTK (49°) in the sagittal plane, with PJA increased to 13° and TK increased to 35°. **f** At 4-year follow-up, GTK increased to 55°, TK increased to 43° and PJA increased to 21°; 14 degrees of increasing PJK angle was measured. TIA remained stable

**Fig. 4:**
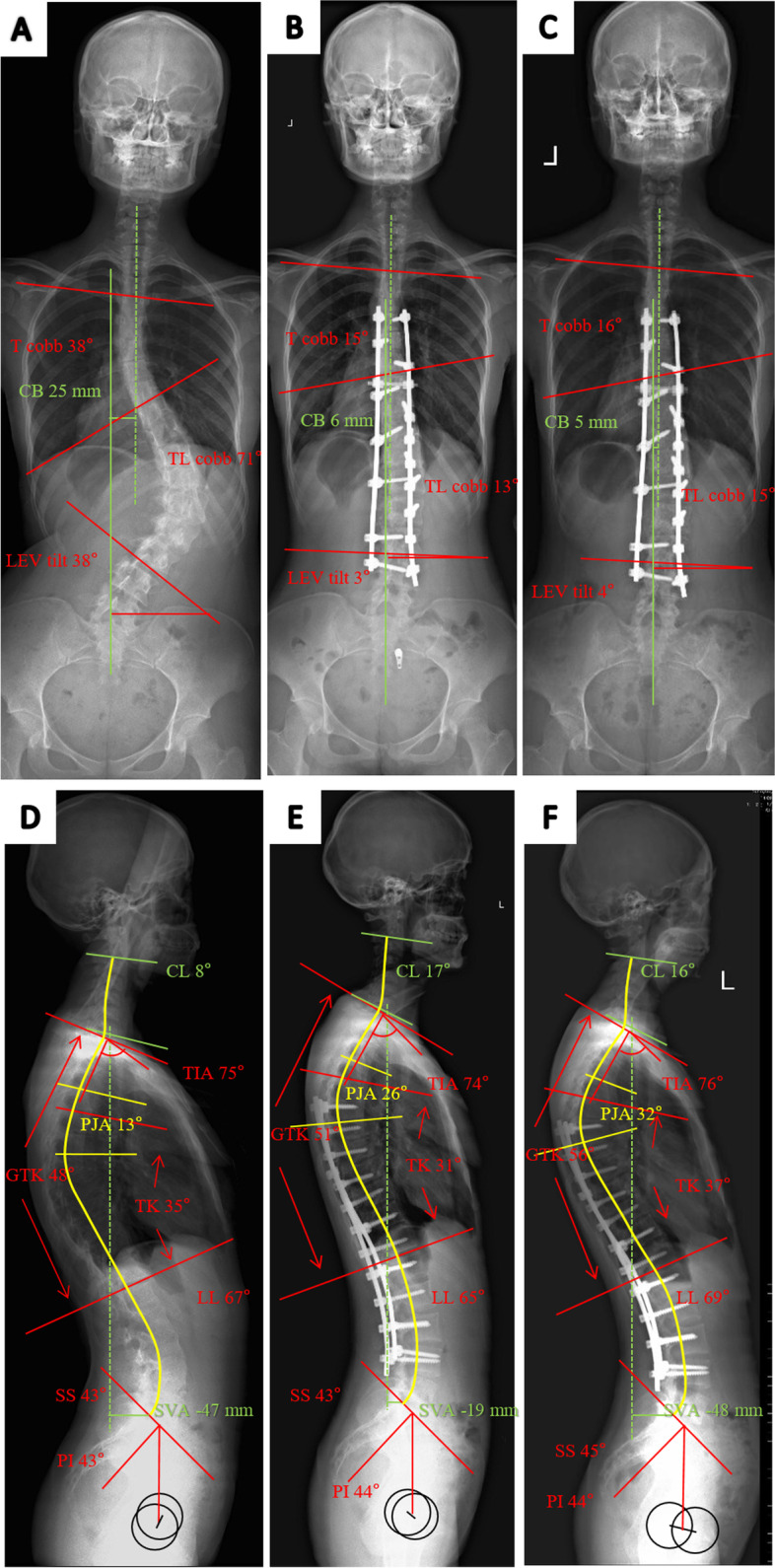
A. Standing PA of a 18-year-old adolescent female. The structural lumbar curve was 71° before surgery. **a** Preoperative T curve was 36°, reduced to 24° at bending film. **b** The postoperative thoracic curve was corrected to 14° and thoracolumbar curve was corrected to 15° at 3 months after surgery. **c** At 2-year follow-up, the main thoracolumbar curve was 13° and the thoracic curve was 13°. **d** The preoperative sagittal alignment showed a large TIA (75°) and GTK (48°). PJA was 13° and TK was 35°. **e** The GTK increased to 51°, with PJA increased to 26° and TK decreased to 31° at first follow-up. **f** At 2-year follow-up, GTK increased to 56°, TK increased to 37° and PJA increased to 32°; 19 degrees of increasing PJK angle was measured. TIA remained stable

The TIA did not change after surgery or during the 2-year follow-up, it may serve as an appropriate predictor of PJK. According to the ROC curve, a TIA of > 71° was the cutoff point for the occurrence of PJK. A larger preoperative TIA, which predicts larger final GTK, facilitates greater kyphosis of the proximal junction during follow-up (Fig. 3). In the patients who underwent extended fusion of the main thoracic curve, this increase in sagittal kyphosis could only be obtained at the proximal junctional area; thus, the PJA increased (Fig. 4). However, patients with a lower TIA, which indicated lower final GTK and T1 tilt, were much more likely to have final cervical kyphosis [8].

However, a few patients with a large TIA had relatively small preoperative TK and GTK because of the rotation of the thoracic curve. These patients may have developed an immediate increase in TK after surgery, but the large TIA also meant that they needed a much larger GTK at the final follow-up. In Ogura’s study, the increased TK at final follow-up time was also risk factor of PJK [23]. The increasing TK and GTK immediately after surgery were not enough for these patients; the TK and GTK continued to increase during follow-up, leading to PJK at the final follow-up.

**Fig. 5:**
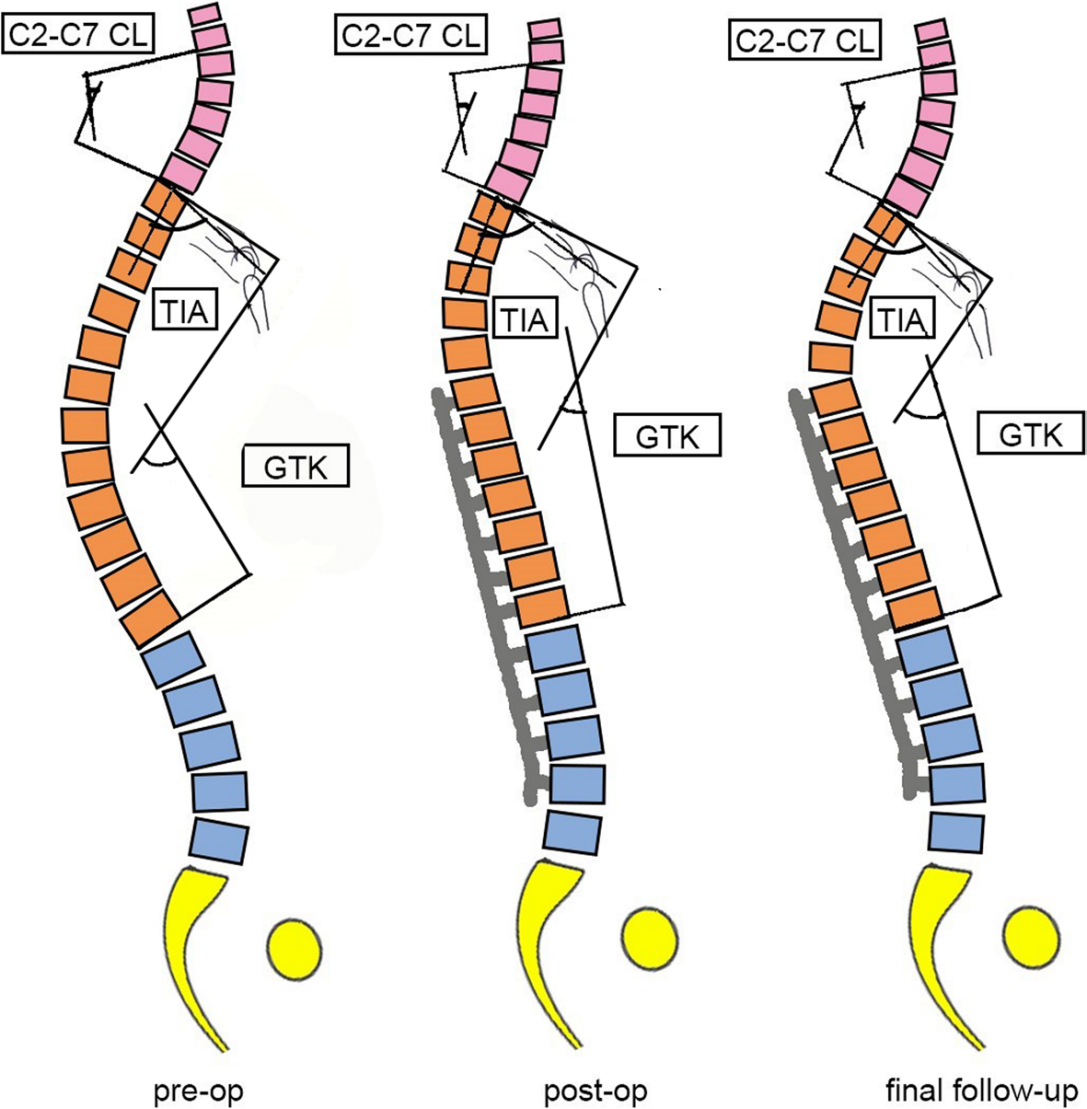
Spontaneous change mechanism of the extending fusion in the patients with a large TIA

A previous study showed that placement of the UIV cephalad to the upper-end vertebra was associated with an increased risk of PJK Lenke 5C patients [4]. Extended fusion provided more correction in the coronal plane with less restoration of TK [15, 16]. In our study, the extended fusion group had a significantly larger immediate reduction in TK, which is reportedly a risk factor for PJK [2, 4, 5]. In this group, the TK decreased immediately after surgery. However, the GTK needed to stay stable with respect to the preoperative GTK, which facilitated the development of PJK in the proximal thoracic region (Fig. 5). Meanwhile, Clément et al. [24] showed that long constructs with insufficient TK is more likely to result in PJK. The extended fusion group also had higher risk of PJK in this study. This may partially explain the higher incidence rate of PJK in the present study rather than the one in previous studies after selective TL/L fusion of Lenke 5C patients [3, 4, 23]. Hence, extended fusion should be carefully evaluated in patients with Lenke 5C AIS who have a TIA of > 71°.

Meanwhile, in patients with an immediate increase in TK, the cause of PJK may also be overcorrection of LL [3]. Hence, larger postoperative LL could lead to an immediate increase in TK [25]. Additionally, the spontaneous change to attain sagittal balance may lead to greater PJK to obtain much larger TK to match the overcorrected LL. The importance of maintaining appropriate postoperative SVA was also found in patients with Lenke 5C AIS [3]. AIS patients with preoperative negative SVA may have higher risk of PJK [23]. However, the present study did not find a correlation between PJK and SVA or LL.

No significant difference was found in the clinical outcome between the PJK and non-PJK groups during the ≥ 2-year follow-up. Furthermore, the domain values for pain, self-image, function, mental health, and satisfaction in SRS-22 scores were good or excellent in both groups. This result is similar to that in a recent study [5]. We also believe that a longer follow-up time is needed to evaluate PJK-induced degeneration and back pain.

The present study had several limitations. First, the number of patients was relatively small for a ≥ 2-year follow-up, and there might not have been enough patients with an abnormal preoperative sagittal profile to detect whether this made a difference in the development of postoperative PJK. Second, TL/L fusion may less accurately reflect the TK than might extended fusion because of the unfused thoracic curve. However, the final Cobb angle of the thoracic curve was not significantly different between the two groups. Third, the accuracy of parameter measurements in patients with PJK remains problematic [20]. Although two surgeons measured and documented the patients’ conditions using an electronic system and calculated the average values, it was still difficult to attain high accuracy.

## Conclusion

Patients with Lenke 5C AIS who underwent posterior fusion with additional thoracic fusion had a higher incidence of PJK than those who underwent TL/L fusion alone. A TIA of > 71° is associated with a higher risk of PJK. Extended fusion should be performed with greater caution in patients with a TIA of > 71°.

## Data Availability

Data will be available upon request to the corresponding author Xi Yang.

## Abbreviations

AIS: Adolescent Idiopathic Scoliosis
LEV: Lower End Vertebra
UEV: Upper End Vertebra
LIV: Lowest Instrumented Vertebra
CSVL: Central Sacral Vertical Line
C7PL: C7 Plumb Line
TL/L: Thoracolumbar/lumbar Curve
T: Thoracic Curve
TK: Thoracic Kyphosis
GTK: Global Thoracic Kyphosis
TIA: Thoracic Inlet Angle
PJA: Proximal Junctional Angle
PJK: Proximal Junctional Kyphosis
LL: Lumbar Lordosis
PI: Pelvic Incidence
PT: Pelvic Tilt
SS: Sacral Slope
SVA: Sagittal Vertical Axis.
SRS-22: Scoliosis Research Society–22 Questionnaire
